# LncGSEA: a versatile tool to infer lncRNA associated pathways from large-scale cancer transcriptome sequencing data

**DOI:** 10.1186/s12864-021-07900-y

**Published:** 2021-07-27

**Authors:** Yanan Ren, Ting-You Wang, Leah C. Anderton, Qi Cao, Rendong Yang

**Affiliations:** 1 The Hormel Institute, University of Minnesota, Austin, MN 55912 USA; 2 Department of Biology, Cedarville University, Cedarville, OH 45314 USA; 3 Department of Urology, Northwestern University Feinberg School of Medicine, Chicago, IL 60611 USA; 4 Robert H. Lurie Comprehensive Cancer Center, Northwestern University Feinberg School of Medicine, Chicago, IL 60611 USA

**Keywords:** Long non-coding RNA, GSEA, Pathway analysis, RNA-seq, TCGA, Cancer transcriptome

## Abstract

**Background:**

Long non-coding RNAs (lncRNAs) are a growing focus in cancer research. Deciphering pathways influenced by lncRNAs is important to understand their role in cancer. Although knock-down or overexpression of lncRNAs followed by gene expression profiling in cancer cell lines are established approaches to address this problem, these experimental data are not available for a majority of the annotated lncRNAs.

**Results:**

As a surrogate, we present lncGSEA, a convenient tool to predict the **lnc**RNA associated pathways through **G**ene **S**et **E**nrichment **A**nalysis of gene expression profiles from large-scale cancer patient samples. We demonstrate that lncGSEA is able to recapitulate lncRNA associated pathways supported by literature and experimental validations in multiple cancer types.

**Conclusions:**

LncGSEA allows researchers to infer lncRNA regulatory pathways directly from clinical samples in oncology. LncGSEA is written in R, and is freely accessible at https://github.com/ylab-hi/lncGSEA.

**Supplementary Information:**

The online version contains supplementary material available at 10.1186/s12864-021-07900-y.

## Background

Advances in sequencing technology and computational algorithms have enabled an unprecedented view of transcriptional landscape of cancer genome, including tens of thousands of lncRNAs being identified. However, the vast majority of annotated lncRNAs remain uncharacterized for their functions in cancer [1]. Mechanistically, lncRNAs can function as oncogenes or tumor suppressors by modulating physiological and pathological processes [2]. The widely-used approach to identify downstream target genes and pathways regulated by a lncRNA is leveraging RNA inference to inhibit the lncRNA expression followed by microarray or RNA-seq gene expression profiling and differential gene expression (DGE) analysis in cancer cell line models [3]. Although this strategy can directly nominate lncRNA associated pathways, laboratory techniques and expenses are required for these *in vitro* experiments and hence most of the detected lncRNAs have no such data available. Additionally, cancer cell lines may not fully resemble cognate tumor profiles in their genomic profiles [4]. Therefore, the clinical relevance of those models has been continuously questioned.

Several bioinformatics tools and webservers (e.g. ncFANs v2.0 [5], lncFunTK [6], AnnoLnc2 [7]) have been developed to identify the enriched gene sets associated with lncRNAs for better understanding of their roles in diverse biological processes and diseases. Existing methods for gene set enrichment analysis (GSEA) typically leverage statistical analyses, such as a hypergeometric test, to evaluate whether a list of user selected genes are enriched in a specific functional gene set, which are classified as over-representation analysis (ORA) and usually need users to define a threshold for gene selection [8]. In contrast, the functional class scoring (FCS) methods calculate the enrichment score utilizing the expression of all genes within a particular gene set. The recent development of KOBAS-i tool demonstrated that FCS is superior than ORA in prioritizing biologically relevant pathways [8]. However, there is a lack of implementations of the FCS method for lncRNA function annotations in cancer research.

To tackle this problem, we developed lncGSEA, a new tool to link gene signatures with a lncRNA expression in tumor patient samples by implementing a fast pre-ranked GSEA method. The fast GSEA method is a typical FCS method, and outperforms the standard GSEA approach in its running time and prediction accuracy [9]. Utilizing the RNA-seq expression data in 33 cancer types including 10,205 tumor samples from the TCGA study [10], lncGSEA is capable of identifying the associated MSigDB [11] or user defined gene sets for over 80,000 annotated lncRNAs. Furthermore, lncGSEA also provides a set of functionalities to enable users to study novel lncRNAs identified by themselves. We applied lncGSEA to predict well-studied lncRNAs in multiple cancers and our prediction accurately revealed the known functional pathways of those lncRNAs. Testing on manually collected lncRNAs with experimental data in cell line models demonstrated that lncGSEA was able to accurately predict these cell line-derived pathways.

## Implementation

### Data processing and workflow

**Fig. 1:**
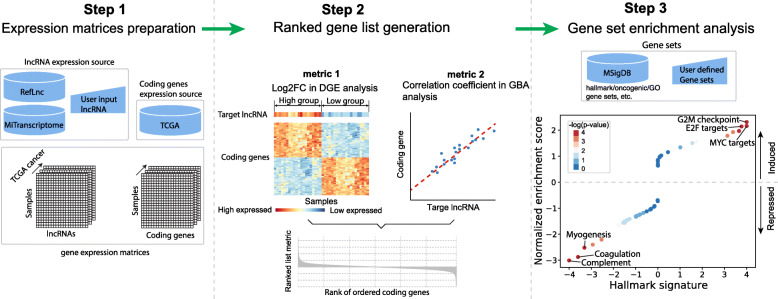
LncGSEA workflow for lncRNA associated pathways detection

LncGSEA follows three steps to infer pathways associated with a lncRNA as illustrated in Fig. 1.

First, lncGSEA prepares the input of expression matrices for lncRNA and coding genes. The expression matrices of lncRNAs represent the expression values of lncRNAs in TCGA tumors. Two databases were used to extract the lncRNA expression values in tumor samples, which include RefLnc [12] and MiTransriptome [13]. RefLnc contained 77,900 lncRNAs with their expression quantified in 18 cancer types. MiTransriptome annotated 58,648 lncRNAs and 8,000 of them were quantified by their expression in 27 cancer types. Users have the option to provide their own lncRNA expression profiles as input for predictions. In addition to the lncRNA expression matrix, lncGSEA obtained the expression profiles of coding genes in 33 cancer types of 10,205 patients from the TCGA study.

Second, lncGSEA will generate a list of ranked genes with two metrics. One approach to rank the genes is to divide patients in each cancer type (e.g. breast, prostate) into two groups with high (top quantile) and low (bottom quantile) expression of the target lncRNA. Next, DGE analysis is performed between the two groups of patients to determine the log2 fold change of each gene as the ranking metric. This approach mimics the lncRNA inhibition or overexpression treatment compared with control conditions in cell line experiments. In addition to DGE method, lncGSEA implements another analysis termed guilt by association (GBA) to construct an expression correlation matrix of the target lncRNA and coding genes [13]. Briefly, the expression levels of the target lncRNA are correlated (Pearson or Spearman) to the expression of all protein-coding genes across all samples in a cancer type. The coding genes are then ranked by the correlation coefficients.

In the third step, the ranked gene list is processed by fast GSEA [9] against a collection of cancer associated gene sets from MSigDB (e.g. hallmark gene sets, oncogenic pathways). The output is a matrix of the association of the target lncRNA with each gene set. Significant associated pathways (FDR q-value < 0.05) can be highlighted and visualized with customized plots (Fig. 1).

## Results

### Evaluation of lncRNA associated pathway prediction with experimental evidence

Through literature search and database mining (Supplementary Methods), we selected eight lncRNAs with known roles in human cancer to analyze using lncGSEA, as summarized in Supplementary Table S1. These lncRNAs have been investigated with *in vitro* or *in vivo* experimental techniques to identify their cancer-related mechanisms or functions. We used 50 MSigDB hallmark gene sets to infer the associated pathways for each of these lncRNAs (Supplementary Methods). The performance of lncGSEA predictions were assessed with four evaluation criteria as described below:

First, we compared the consistency of predictions with different gene ranking approaches. We quantified the similarity between the two ranked gene lists by the DGE and GBA approaches (Supplementary Methods). In a meta-analysis of the eight lncRNAs, we found significant similarity (meta *p*-value < 0.001) between the two ranked gene lists (Supplementary Table S2). Furthermore, we compared the running time between the two methods. Our testing indicated that GBA method was more than 30 times faster than DGE (Supplementary Figure S1).

Next, we examined the agreement of predicted pathways between RefLnc and MiTranscriptome. All of the eight lncRNAs have been annotated by both of the two databases. We extracted the lncRNA expression values of the TCGA patients from each database and made predictions separately. We observed robust predictions as reflected by the order of each lncRNA associated gene sets ranked by normalized enrichment scores (Supplementary Table S3).

Third, we sought to test whether lncGSEA can identify pathways inferred directly from lncRNA knock-down experiments. In our list, three lncRNAs, including EPIC1, SBF2-AS1 and DNM3OS, have RNA-seq data for wild-type (WT) and lncRNA knock-down (KD) from the corresponding cancer cell line models. We performed DGE analysis between WT and KD, and identified the enriched hallmark gene sets for each of the three lncRNAs (Supplementary Methods). When comparing the cell line experiment-derived hallmark gene sets with lncGSEA predictions, we observed significant similarity (meta *p*-value < 0.001) between them (Supplementary Table S4), suggesting lncGSEA is able to predict pathways derived from *in vitro* experiments.

**Fig. 2:**
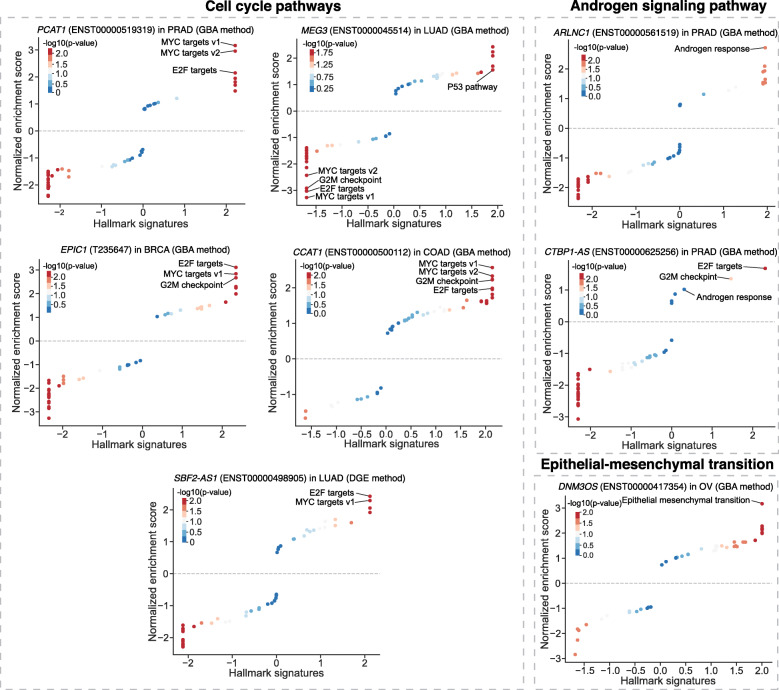
LncGSEA predicted pathways that are associated with eight well-studied lncRNAs in cancer. GSEA was used to test for enrichment of MSigDB hallmark gene sets in TCGA tumor gene expression data ordered based on differential expression genes (DGE) or guilt by association (GBA) methods in breast (BRCA), colon (COAD), lung (LUAD), ovarian (OV) and prostate (PRAD) cancer. Cancer-hallmark signature genes were plotted based on their normalized enrichment score

Finally, we compared lncGSEA predictions with reported pathways of each lncRNA from the original studies. We demonstrated that lncGSEA is capable of nominating these lncRNA associated pathways, such as androgen signaling for ARLNC1 and CTBP1-AS, cell cycle pathways (e.g. MYC, E2F, G2M and P53) for PCAT-1, EPIC1, MEG3, CCAT-1 and SBF2-AS1, and epithelial–mesenchymal transition for DNM3OS (Fig. 2).

### Comparison with existing tools

We further compared the performance of lncGSEA with two recently developed lncRNA annotation tools, ncFANs v2.0 and AnnoLnc2, for predicting the pathways or gene sets associated with the eight lncRNAs of known functions (Supplementary Methods). Given our observations that lncGSEA successfully predicted the known associated pathways of these lncRNAs (Fig. 2), we sought to evaluate whether the two existing tools can accurately annotate the functions of these lncRNAs. As a result, we found ncFANs v2.0 was able to predict the hallmark gene sets for seven lncRNAs, but failed to return any results for CCAT1. Among the seven lncRNAs, ncFANs v2.0 did not successfully nominate the known associated pathways for three lncRNAs including MEG3, ARLNC1 and CEBP1-AS1 in its top ranked predictions (Supplementary Table S5). Next, we applied AnnoLnc2 to annotate the eight lncRNAs for enriched GO terms of biological processes. We found half of these lncRNAs were not annotated by AnnoLnc2 and only the lncRNA DNM3OS of the four annotated lncRNAs returned the GO terms (e.g. extracellular matrix organization) that reflect the literature-supported functions (i.e. epithelial–mesenchymal transition) (Supplementary Table S6). Taken together, our results suggest lncGSEA outperforms existing lncRNA annotation tools for functional predictions of lncRNAs in oncology.

## Discussion

In this study, we present the lncGSEA software that is implemented in R language and easily installed for users in cancer research to explore the functional pathways that are associated with lncRNAs. LncGSEA allows users to choose two types of ranking metrics, correlation of coefficient (GBA option) and log2 fold change (DGE option) for calculating the enriched genes sets with GESA analysis. As showed in our results, the two ranking metrics generally provide consistent predictions. We also highlighted that GBA approach tended to run faster than DGE method. However, certain lncRNAs’ expression can be highly heterogenous in a specific cancer type (e.g. no expression at all in some of the cancer samples but highly expressed in the other part of cancer samples). In this situation, we would recommend to use the DGE method that separates cancer samples into two groups (low vs. high) based on the lncRNA’s expression and then use log2 fold change to rank the genes for downstream enrichment analysis. In an effort to allow users easily access the lncGSEA predictions, we developed a web interface presenting the predicted MsigDB hallmark gene sets associated with MiTranscriptome or RefLnc annotated lncRNAs in diverse cancer types from the TCGA study (https://ylab-hi.shinyapps.io/lncgsea_app/). We will continuously update the web server based on users’ feedback.

LncGSEA adopts the conventional GSEA method to conduct pathway-level association with lncRNAs. Because gene members in a pathway may have distinct functional roles and regulatory relationships among them, identifying key gene drivers in each enriched pathway that are associated with the lncRNA would be a useful feature to be added. We have enabled lncGSEA to report the leading-edge genes contributing the most to the enrichment score of each pathway, which can be used as the potential gene targets of the lncRNA. Future work is needed to include dedicated approaches designed for the identification at both pathway level and gene level, which is termed as bilevel selection [14]. For example, a Bayesian model developed by Jiang and colleagues [14] that integrates the gene pathway and interactions information could complement the GSEA approach for pathway analysis and key gene signature discovery in the further development of lncGSEA package.

## Conclusions

We presented lncGSEA as a robust tool for detecting lncRNA regulatory pathways from large-scale tumor tissue transcriptome sequencing data. We demonstrated that lncGSEA reliably detects known lncRNA associated gene signatures with literature and experimental evidence support. We anticipate lncGSEA to be a powerful tool for better understanding the functional mechanisms and clinical relevance of currently understudied lncRNAs in human cancers.

### Availability of and requirements

Project name: LncGSEA.

Project home page: https://github.com/ylab-hi/lncGSEA.

Operating system(s): platform independent.

Programming language: R.

Other requirements: R 3.6.1 or higher.

License: MIT.

Any restrictions to use by non-academics: license needed.

## Supplementary Information

**Additional file 1: Supplementary Methods.** **Supplementary Table S1. **Selected LncRNAs for lncGSEA analysis. **Supplementary Table S2.** Comparing two DGE and GBA as metrics for ranked gene list. lncRNA expressions in TCGA study are based on RefLnc annotations. Order direction, weighted overlap scores and statistical significance of two ranked coding gene list were calculated by OrderedList.** Supplementary Table S3. **Calculated similarity of 50 ordered MSigDB Hallmark gene sets for each lncRNA based on RefLnc or MiTranscriptome annotations. Gene sets are ranked by normalized enrichment scores. Predictions of associated gene sets are based on GBA ranking approach. **Supplementary Table S4. **Calculated similarity of 50 ordered MSigDB Hallmark gene sets from lncGSEA and cancer cell line-derived pathway predictions. **Supplementary Figure S1.** Running time comparison of using GBA and DGE metrics to predict lncRNA CCAT1 associated hallmark pathways in COAD cohort of TCGA. Both metrics were evaluated 10 times, and their running time statistics and graph were first calculated by *microbenchmark (times = 10)* in “microbenchmark” and then plotted by *autoplot* in “ggplot2”. The task was done on MacOS with a memory size of 8 GB 1600 MHz DDR3 and a processor of 1.8 GHz Intel Core i5.

**Additional file 2: Supplementary Table S5.** Top 5 enriched hallmark pathways of 7 lncRNAs predicted by lncGSEA and ncFANs v2.0. **Supplementary Table S6.** Top 5 enriched GO:BP pathways of 4 lncRNAs predicted by lncGSEA and AnnoLnc2.

## Data Availability

Gene expression data of TCGA samples are available at Genomic Data Commons Data Portal [15] (dbGaP study accession number: phs000178).

## Abbreviations

LncRNA: Long non-coding RNA
GSEA: Gene Set Enrichment Analysis
ORA: Over-Representation Analysis
FCS: Functional Class Scoring
DGE: Differential Gene Expression
GBA: Guilt by Association
FDR: False Discovery Rate
KD: Knock Down
WT: Wild Type
TCGA: The Cancer Genome Atlas
