# Supplementary material for: LncGSEA: a versatile tool to infer lncRNA associated pathways from large-scale cancer transcriptome sequencing data
Source: BMC Genomics. 2021 Jul 27;22:574. doi: 10.1186/s12864-021-07900-y (PMC8314497; doi:10.1186/s12864-021-07900-y)
Supplement: Supplementary file 1 — Additional file 1: Supplementary Methods. Supplementary Table S1. Selected LncRNAs for lncGSEA analysis. Supplementary Table S2. Comparing two DGE and GBA as metrics for ranked gene list. lncRNA expressions in TCGA study are based on RefLnc annotations. Order direction, weighted overlap scores and statistical significance of two ranked coding gene list were calculated by OrderedList. Supplementary Table S3. Calculated similarity of 50 ordered MSigDB Hallmark gene sets for each lncRNA based on RefLnc or MiTranscriptome annotations. Gene sets are ranked by normalized enrichment scores. Predictions of associated gene sets are based on GBA ranking approach. Supplementary Table S4. Calculated similarity of 50 ordered MSigDB Hallmark gene sets from lncGSEA and cancer cell line-derived pathway predictions. Supplementary Figure S1. Running time comparison of using GBA and DGE metrics to predict lncRNA CCAT1 associated hallmark pathways in COAD cohort of TCGA. Both metrics were evaluated 10 times, and their running time statistics and graph were first calculated by microbenchmark (times = 10) in “microbenchmark” and then plotted by autoplot in “ggplot2”. The task was done on MacOS with a memory size of 8 GB 1600 MHz DDR3 and a processor of 1.8 GHz Intel Core i5. [file 12864_2021_7900_MOESM1_ESM.docx]

| Supplementary Information  LncGSEA: a versatile tool to infer lncRNA associated pathways from large-scale cancer transcriptome sequencing data  Yanan Ren^1^, Ting-You Wang^1^, Leah C. Anderton^2^, Qi Cao^3,4^ and Rendong Yang^1,*^  ^1^The Hormel Institute, University of Minnesota, Austin, MN 55912, ^2^Department of Biology, Cedarville University, Cedarville, OH 45314, ^3^Department of Urology, ^4^Robert H. Lurie Comprehensive Cancer Center, Northwestern University Feinberg School of Medicine, Chicago, IL, 60611. *To whom correspondence should be addressed. |
| --- |

**1. Introduction:**

This document contains supplementary information for the article *lncGSEA: a versatile tool to infer lncRNA associated pathways from large-scale cancer transcriptome sequencing data.*

**2. Supplementary Methods:**

*Predicting associated MSigDB gene sets for selected lncRNAs*

We collected lncRNA candidates from Table 1 of (Slack and Chinnaiyan, 2019) and lncRNA2Target v2.0 database (Cheng *et al.*, 2019). We manually reviewed each of the listed lncRNAs in their original studies and selected eight lncRNAs for further testing as these candidates have been well-studied with experimentally confirmed downstream pathways. We predicted each lncRNA associated pathways against a collection of MSigDB gene sets including 50 hallmark gene sets (H), which represent a wide range of biological processes and diseases (Liberzon *et al.*, 2015).

*Calculating similarity of ordered genes or gene sets lists*

To compare the similarity of two ranked gene lists generated by DEG and GBA method respectively, we utilized the OrderedList, a Bioconductor package to quantify the similarity between ordered list (Lottaz *et al.*, 2006). We ranked the gene list by Log2 fold change for DEG method and correlation coefficient for GBA method. To measure the similarity of two ordered MSigDB gene set lists from two predicting approaches (e.g. RefLnc vs. MiTranscriptome, TCGA Patients vs. cancer cell line models), we ranked the gene sets by the normalized enrichment score given by the gene set enrichment analysis (GSEA). Empirical P-values of similarity are corrected by Benjamini-Hochberg method for multiple comparison. Meta-analysis of results across lncRNAs was conducted using the Edgington method implemented by sump function in “metap” R package of combining P-values from independent tests.

*DEG and pathway analysis for lncRNA knock-down experiments*

In our lncRNA list as showed in Supplementary Table S1, EPIC1, SBF2-AS1 and DNM3OS have RNA-seq data available for lncRNA and non-specific control knock-down experiments. We downloaded the processed RNA-seq read count data from the GEO database with accession number listed in Supplementary Table S4. For each lncRNA, we applied DESeq2 (Love *et al.*, 2014) to calculate the log2 fold change and statistical significance of coding genes differentially expressed between the knock-down and control conditions. We further ranked the gene list by log2 fold change and performed GSEA analysis using the MSigDB hallmark gene sets.

*Functional annotation of lncRNAs by ncFANs v2.0 and AnnoLnc2*

For each lncRNA, we use their official names as input to run ncFANs v2.0 with the default cutoff 0.4 and the cancer-specific option for both co-expression and co-methylation network. For example, we selected TCGA-COAD when we do functional analysis for CCAT1. Default parameters of lncRnet and RF-based network were used in the analysis. After four individual network analyses finished, a merged network was generated by selecting “merge” button. The functional annotation of the merged network using MsigDB hallmark pathways was downloaded for comparison with the results of lncGSEA. AnnoLnc2 requires lncRNA’s fasta sequence as input. We obtained the corresponding sequence of each lncRNA from the UCSC genome browser for AnnoLnc2 predictions. We used the function annotations by GO terms of biological processes for comparison with those predicted by lncGSEA.

**3. Supplementary Tables:**

**Supplementary Table S1. Selected LncRNAs for lncGSEA analysis**

| LncRNA name | Cancer type examined | *In vitro* experimental technology | Cancer-related mechanisms | Reference |
| --- | --- | --- | --- | --- |
| EPIC1 | Breast (BRCA) | siRNA knockdown | Promote MYC/E2F/Cell cycle pathway | (Wang *et al.*, 2018) |
| CCAT1 | Colon (COAD) | siRNA knockdown | Promote MYC/Cell cycle pathway | (Kim *et al.*, 2014) |
| MEG3 | Lung (LUAD) | Overexpression | Increase P53 levels and downregulate MYC pathway | (Yan-hua *et al.*, 2015; Lu *et al.*, 2013) |
| SBF2-AS1 | Lung (LUAD) | siRNA knockdown | Increase E2F1 expression and upregulate cell cycle pathway | (Chen *et al.*, 2019) |
| DNM3OS | Ovarian (OV) | siRNA knockdown | Upregulate epithelial-to-mesenchymal transition | (Mitra *et al.*, 2017) |
| ARLNC1 | Prostate (PRAD) | siRNA knockdown | Upregulate AR signaling | (Zhang *et al.*, 2018) |
| CTBP1-AS | Prostate (PRAD) | siRNA knockdown | Activate AR signaling | (Takayama *et al.*, 2013) |
| PCAT1 | Prostate (PRAD) | siRNA knockdown | Promote cell proliferation through cMyc | (Prensner *et al.*, 2014) |

**Supplementary Table S2. Comparing two DGE and GBA as metrics for ranked gene list.** lncRNA expressions in TCGA study are based on RefLnc annotations. Order direction, weighted overlap scores and statistical significance of two ranked coding gene list were calculated by OrderedList.

| LncRNAs | Order | Similarity scores | Adj. P-values | |  |
| --- | --- | --- | --- | --- | --- |
| EPIC1 | Direct | 9230.0 | | 0.000 | |
| CCAT1 | Direct | 536.0 | | 0.043 | |
| MEG3 | Direct | 14701.0 | | 0.000 | |
| SBF2-AS1 | Direct | 7930.0 | | 0.000 | |
| DNM3OS | Direct | 19019.0 | | 0.000 | |
| ARLNC1 | Direct | 24371.0 | | 0.000 | |
| CTBP1-AS | Direct | 25577.0 | | 0.000 | |
| PCAT1 | Direct | 13275.0 | | 0.000 | |

DGE, differential gene expression; GBA, guilt by association.

**Supplementary Table S3. Calculated similarity of 50 ordered MSigDB Hallmark gene sets for each lncRNA based on RefLnc or MiTranscriptome annotations.** Gene sets are ranked by normalized enrichment scores. Predictions of associated gene sets are based on GBA ranking approach.

| LncRNAs | RefLnc ID | MiTranscriptome ID | | Order | Weighted overlap scores | Adj. P-values |
| --- | --- | --- | --- | --- | --- | --- |
| EPIC1 | ENST00000426452 | | T235647 | Direct | 22.5 | 0.000 |
| CCAT1 | ENST00000500112 | | T351192 | Direct | 22.6 | 0.000 |
| MEG3 | ENST00000452514 | | T110324 | Direct | 23.8 | 0.000 |
| SBF2-AS1 | ENST00000498905 | | T054676 | Direct | 19.2 | 0.000 |
| DNM3OS | ENST00000417354 | | T025160 | Direct | 25.5 | 0.000 |
| ARLNC1 | ENST00000561519 | | T136805 | Direct | 19.9 | 0.000 |
| CTBP1-AS | ENST00000625256 | | T259327 | Direct | 15.6 | 0.000 |
| PCAT1 | ENST00000519319 | | T351126 | Direct | 26.7 | 0.000 |

**Supplementary Table S4. Calculated similarity of 50 ordered MSigDB Hallmark gene sets from lncGSEA and cancer cell line-derived pathway predictions.**

| LncRNAs | Cell line used | GEO accession ID | Order | Weighted overlap scores | Adj. P-values | | |
| --- | --- | --- | --- | --- | --- | --- | --- |
| EPIC1 | MCF7 | GSE98538 | Reverse | 14.7 | | 0.000 |  |
| SBF2-AS1 | A549 | GSE103016 | Reverse | 16.4 | | 0.000 |  |
| DNM3OS | SK-OV-3 | GSE104295 | Reverse | 8.2 | | 0.161 |  |

**4. Supplementary Figures:**

**Supplementary Figure S1**. Running time comparison of using GBA and DGE metrics to predict lncRNA CCAT1 associated hallmark pathways in COAD cohort of TCGA. Both metrics were evaluated 10 times, and their running time statistics and graph were first calculated by *microbenchmark (times = 10)* in “microbenchmark” and then plotted by *autoplot* in “ggplot2”. The task was done on MacOS with a memory size of 8 GB 1600 MHz DDR3 and a processor of 1.8 GHz Intel Core i5.

**Reference:**

Chen,R. *et al.* (2019) Long Noncoding RNA SBF2-AS1 Is Critical for Tumorigenesis of Early-Stage Lung Adenocarcinoma. *Mol. Ther. - Nucleic Acids*, **16**, 543–553.

Cheng,L. *et al.* (2019) LncRNA2Target v2.0: a comprehensive database for target genes of lncRNAs in human and mouse. *Nucleic Acids Res.*, **47**, D140–D144.

Kim,T. *et al.* (2014) Long-range interaction and correlation between MYC enhancer and oncogenic long noncoding RNA CARLo-5. *Proc. Natl. Acad. Sci.*, **111**, 4173–4178.

Liberzon,A. *et al.* (2015) The Molecular Signatures Database (MSigDB) hallmark gene set collection. *Cell Syst.*, **1**, 417–425.

Lottaz,C. *et al.* (2006) OrderedList--a bioconductor package for detecting similarity in ordered gene lists. *Bioinformatics*, **22**, 2315–2316.

Love,M.I. *et al.* (2014) Moderated estimation of fold change and dispersion for RNA-seq data with DESeq2. *Genome Biol.*, **15**, 550.

Lu,K. *et al.* (2013) Long non-coding RNA MEG3 inhibits NSCLC cells proliferation and induces apoptosis by affecting p53 expression. *BMC Cancer*, **13**, 461.

Mitra,R. *et al.* (2017) Decoding critical long non-coding RNA in ovarian cancer epithelial-to-mesenchymal transition. *Nat. Commun.*, **8**, 1604.

Prensner,J.R. *et al.* (2014) The Long Non-Coding RNA PCAT-1 Promotes Prostate Cancer Cell Proliferation through cMyc. *Neoplasia*, **16**, 900–908.

Slack,F.J. and Chinnaiyan,A.M. (2019) The Role of Non-coding RNAs in Oncology. *Cell*, **179**, 1033–1055.

Takayama,K. *et al.* (2013) Androgen-responsive long noncoding RNA CTBP1-AS promotes prostate cancer. *EMBO J.*, **32**, 1665–1680.

Wang,Z. *et al.* (2018) lncRNA Epigenetic Landscape Analysis Identifies EPIC1 as an Oncogenic lncRNA that Interacts with MYC and Promotes Cell-Cycle Progression in Cancer. *Cancer Cell*, **33**, 706-720.e9.

Yan-hua,L. *et al.* (2015) Long noncoding ribonucleic acids maternally expressed gene 3 inhibits lung cancer tumor progression through downregulation of MYC. *Indian J. Cancer*, **52**, 190.

Zhang,Y. *et al.* (2018) Analysis of the androgen receptor–regulated lncRNA landscape identifies a role for ARLNC1 in prostate cancer progression. *Nat. Genet.*, **50**, 814–824.
